# Comparative Analysis for the Presence of IgG Anti-Aquaporin-1 in Patients with NMO-Spectrum Disorders

**DOI:** 10.3390/ijms17081195

**Published:** 2016-07-23

**Authors:** Ismael Sánchez Gomar, María Díaz Sánchez, Antonio José Uclés Sánchez, José Luis Casado Chocán, Nela Suárez-Luna, Reposo Ramírez-Lorca, Javier Villadiego, Juan José Toledo-Aral, Miriam Echevarría

**Affiliations:** 1 Instituto de Biomedicina de Sevilla (IBiS), Hospital Universitario Virgen del Rocío/CSIC/Universidad de Sevilla, Seville 41013, Spain; ismael_sg84@hotmail.com (I.S.G.); neluqui@yahoo.es (N.S.-L.); reporamirez@us.es (R.R.-L.); fvilladiego@us.es (J.V.); juanjo@us.es (J.J.T.-A.); 2 Unidad de Gestión Clínica de Neurociencias, Servicio de Neurología del Hospital Universitario Virgen del Rocío, Seville 41013, Spain; mariadiazsanchez@hotmail.com (M.D.S.); antonioj.ucles@gmail.com (A.J.U.S.); jcasadoch@gmail.com (J.L.C.C.); 3 Centro de Investigación Biomédica en Red sobre Enfermedades Neurodegenerativas (CIBERNED), Madrid 28029, Spain; 4 Centro de Investigación Biomédica en Red sobre Enfermedades Respiratorias (CIBERES), Madrid 28029, Spain

**Keywords:** AQP1, AQP4, NMO-IgG, HEK cells, neuromyelitis optica

## Abstract

Detection of IgG anti-Aquaporin-4 (AQP4) in serum of patients with Neuromyelitis optica syndrome disorders (NMOSD) has improved diagnosis of these processes and differentiation from Multiple sclerosis (MS). Recent findings also claim that a subgroup of patients with NMOSD, serum negative for IgG-anti-AQP4, present antibodies anti-AQP1 instead. Explore the presence of IgG-anti-AQP1 using a previously developed cell-based assay (CBA) highly sensitive to IgG-anti-AQP4. Serum of 205 patients diagnosed as NMOSD (8), multiple sclerosis (94), optic neuritis (39), idiopathic myelitis (29), other idiopathic demyelinating disorders of the central nervous system (9), other neurological diseases (18) and healthy controls (8), were used in a CBA over fixed HEK cells transfected with hAQP1-EGFP or hM23-AQP4-EGFP, treated with Triton X-100 and untreated. ELISA was also performed. Analysis of serum with our CBA indicated absence of anti-AQP1 antibodies, whereas in cells pretreated with detergent, noisy signal made reliable detection impossible. ELISA showed positive results in few serums. The low number of NMOSD serums included in our study reduces its power to conclude the specificity of AQP1 antibodies as new biomarkers of NMOSD. Our study does not sustain detection of anti-AQP1 in serum of NMOSD patients but further experiments are expected.

## 1. Introduction

Neuromyelitis optica (NMO) is an autoimmune, inflammatory and demyelinating disease of the central nervous system (CNS) that primarily affects the optic nerves and spinal cord. Patients with this disease, also known as Devic syndrome, present recurrent episodes of optic neuritis and acute myelitis that conduce to a loss of uni- or bilateral vision and to a sensitive and motor compromise below the affected medullar level with frequent loss of sphincters control [1,2]. Although it was considered for a long time as a variant of multiple sclerosis (MS), new pathological and serological tests have contributed to identify this disorder as a different disease [3]. The main evidence for such distinction was provided by Lennon et al. [3,4,5] when they discovered the presence of specific immunoglobulins (IgG-NMO) in serum of NMO patients, IgG that were usually absent in classical forms of MS. The antigen recognized for IgG-NMO is Aquaporin-4 (AQP4), the most abundantly expressed aquaporin in the CNS [6,7,8], highly localized in astrocytes membrane facing blood vessels, in ependymal cells of brain ventricles, and layers of the meninges surrounding the brain and spinal cord [4]. Recent works present convincing evidences supporting a direct involvement of AQP4 autoantibodies (IgG-NMO) in the development of NMO disease [4,9,10,11]. These antibodies have also been identified in patients with limited forms of this disease (recurrent idiopathic optic neuritis, etc.), but with a high risk of suffering subsequent clinical episodes. In this sense, last year, the International Panel for NMO diagnosis was convened to develop revised diagnostic criteria to include these limited forms of the disease in order to facilitate a diagnosis consensus. The new nomenclature defines the unifying term NMO spectrum disorder (NMOSD) that is stratified further by the presence or absence of IgG-NMO and the presence of an isolated or several clinical events [12]. Unfortunately, little more than 20% of patients included in the group of demyelinating disorders of the NMOSD are seronegative for anti-AQP4 antibodies [12,13]. Besides AQP4, human astrocytes in the CNS also express Aquaporin-1 (AQP1), and specifically a large expression of this protein has been detected in areas with proclivity to develop NMO-like lesions as in the spinal cord, optic nerves and in the white matter of the brain [14,15,16,17]. In the last few years, several groups [17,18,19,20] have actively searched for the presence of anti-AQP1 antibodies in the serum of patients with chronic demyelinating process of the CNS using different methods, and some conflicting findings have raised in this respect. On one side, two groups [17,18,19], one based in protein immunobloting and ELISA [17,18] and the other in results obtained from a cell based assay (CBA) using fixed HEK cells pretreated with triton [19], have demonstrated that a subgroup of patients, some of them seronegative for anti-AQP4, present antibodies anti AQP1 in serum. Such results allowed these authors to suggest that this antibody may be taken as another new classifying biomarker for these demyelinating disorders. In contrast to this, Schanda et al., [20] failed to confirm the presence of AQP1 antibodies in NMOSD patients using a live cell immunofluorescence assay. Therefore, given this scenario, we decided to explore in a cohort of 205 serums, between healthy and patients, recruited from the Neurology Service of Hospital Universitario Virgen del Rocío, the biggest hospital of Andalusia, Spain; for the presence of anti-AQP1 antibodies, with the aim of helping to establish a simple assay to identify with specificity the presence of these antibodies and contribute to clarify whether or not AQP1-antibody, should be considered as a new protagonist for the origin of NMOSD pathology.

In the present work we show the results obtained using a protocol of fixed CBA with HEK cells, as published in our previous work [21], to identify presence of anti-AQP4 antibodies, after adapting the protocol in order to look for AQP1 expression as well. Serum of patients were analyzed: 8 NMOSD, 94 MS, 39 optic neuritis (ON), 29 idiopathic myelitis, 9 other idiopathic demyelinating processes of CNS, 18 other neurological disorders and 8 controls, and the assay was performed over AQP1- or AQP4-transfected HEK293 cells, either permeabilized with triton X-100 or untreated. We conclude from our results that the CBA as described in the present work or by others [20,21] does not give a specific signal that can allow for the detection of anti-AQP1 antibodies in the serum patients. However, the low number of NMOSD serums included in our analysis reduces the strength of more definitive conclusions, and we think that it would be still worth to dedicate some additional efforts to clarify whether or not anti-AQP1 antibodies are present in serum of patients, and if so, whether the presence of these antibodies may be used as a new biomarker, that could be widely used as a complement to the anti-AQP4 antibody assay that still remains as a paradigm biomarker of NMOSD diagnosis.

## 2. Results and Discussion

Recent works have provided convincing evidences that implicate the direct participation of anti-AQP4 antibodies in the pathogenesis and diagnosis of the NMOSD [9,10,11]. Detection of IgG-anti-AQP4 in the serum of patients has become a decisive biomarker for diagnosis of most NMOSD patients but unfortunately still around 20% of NMOSD patients result in serum-negative for these antibodies [12,13]. Finding another biomarker for these disorders in these NMO-AQP4 serum negative patients would be highly desirable and has led investigators to turn their attention to AQP1, another protein of the same family of integral membrane proteins, whose expression has also been observed in the CNS in a natural way, but it is, fascinatingly, overexpressed when injury of the nervous tissue occurs [14,15,16]. Two previous works, Tzartos et al., [17] and Long et al., [19], indicated that anti-AQP1 antibodies are present in the serum of patients with NMOSD. We decided therefore to explore the presence of these antibodies in the serum of a large repertoire of patients for which detection of IgG-anti-AQP4 was previously tested using a method described in our laboratory [21], that was previously validated contrasting our results against those obtained for the same serums, by the only center of reference for diagnosis of anti-NMO antibodies in Spain (Hospital Clínic, Barcelona, Spain).

**Figure 1:**
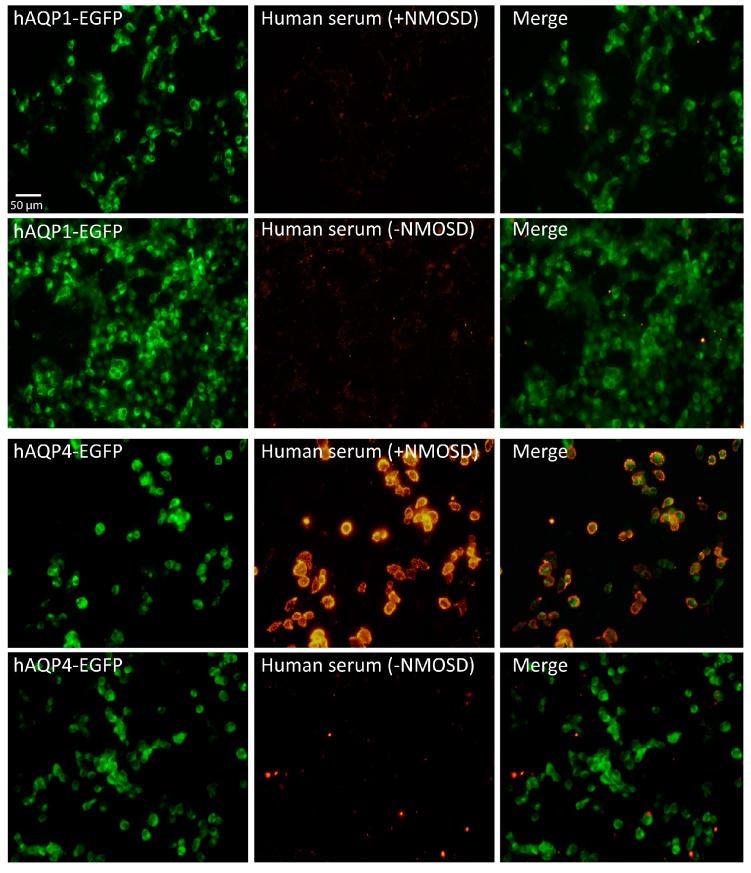
Immunofluorescence assay in HEK cells expressing AQP1 or AQP4 with serum of patients. The fluorescence signal from HEK cells expressing human AQP1 (hAQP1-EGFP) or AQP4 (hAQP4-EGFP) fused to GFP is shown in green (left column). In the central panels the immune reaction produced by serum patients either from the Neuromyelitis optica spectrum disorder (+NMOSD) or not (−NMOSD) over AQP1 expressing cells revealed absence of anti-AQP1 antibodies in analyzed serums. In contrast, serum from a positive anti-AQP4 antibody patient (+NMOSD) showed clear reactivity over AQP4 expressing cells (yellow signal), while negative results are obtained in the absence of IgG anti-AQP4 in a patient (−NMOSD). The merge image of both fluorescent signals is shown in the right column. Scale bar = 50 µm.

We initiated the screening using the same protocol for CBA previously described [21] but now, HEK cells were transfected with human AQP1-EGFP plasmid to produce a large expression of AQP1 in the cell membrane. Readout of AQP1 expression was followed by direct appearance of green fluorescent signal coming from the EGFP fused to AQP1 (Figure 1, hAQP1-EGFP). As shown in Figure 1, total absence of AQP1 antibodies was obtained after analysis of 205 serums obtained from patients and controls (Table 1). Independently of whether the serum came from a patient diagnosed as NMOSD or not, the result was always the same: absence of fluorescent signal.

**Figure 2:**
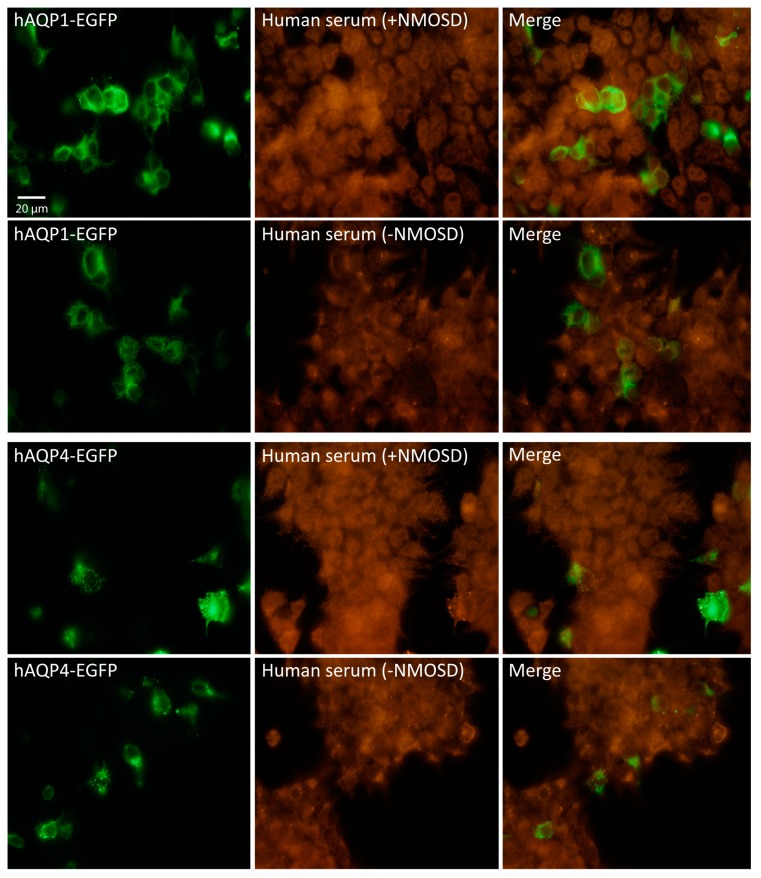
Immunofluorescence assay in HEK cells treated with Triton X-100. Panels are as indicated in Figure 1, but here experiments were done using a protocol of permeabilization with triton X-100 as indicated in the text (material and methods section). High background of fluorescent signal (middle column panels) was detected over all cells in the plate regardless expression or not of AQPs in them, confirming unspecific reaction. Scale bar = 20 µm

The high specificity and sensitivity of the CBA used here was further verified in parallel experiments in which HEK cells were transfected with human AQP4 (Figure 1, hAQP4-EGFP), and by doing so, AQP4 antibodies were clearly detected in the serum of NMOSD patients, while they were never observed in patients with other diseases and healthy controls. Then, assuming that the discrepancy with the results obtained by others [17,18,19] was due to differences in the permeabilization procedure, we treated HEK cells with triton X-100 as indicated by Long et al. [19]. Using their permeabilization protocol, we obtained a large fluorescence signal, but it was highly unspecific, and coming from all cells in the plate regardless of whether they expressed or not AQP1 or AQP4 (Figure 2). These noisy results obtained in our hands with such a procedure led us to conclude that it is not accurate enough for the detection neither of AQP1 nor AQP4 antibodies in the serum of patients.

**Figure 3:**
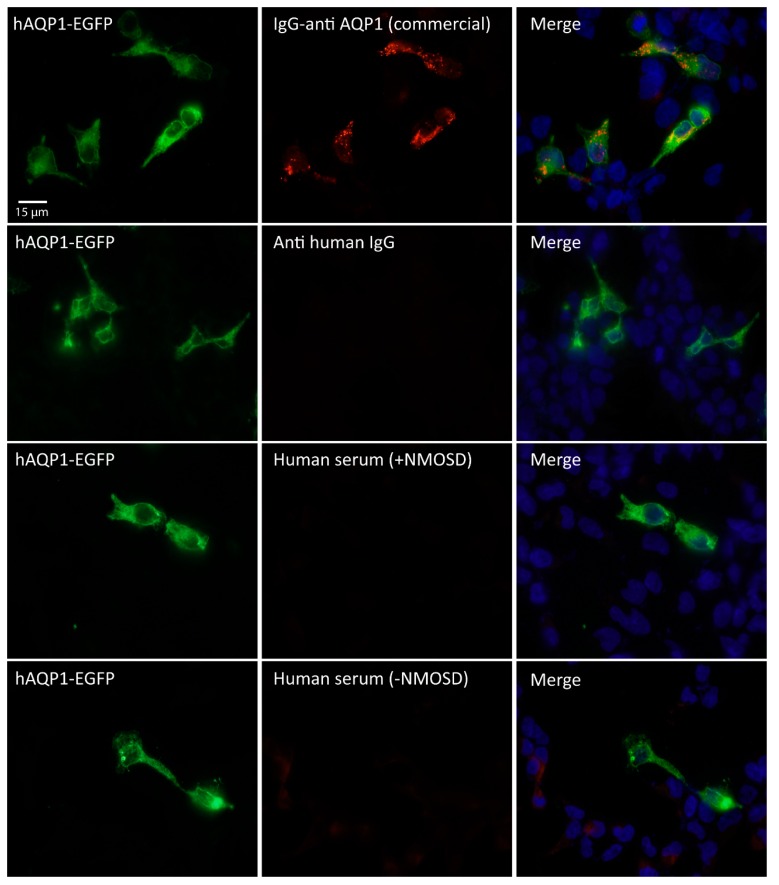
Immunofluorescence assay with commercial antibody for AQP1. HEK cells expressing AQP1 in green (left column) immune reacted with a commercial IgG-anti-AQP1 (top middle panel, red signal), using fixed cells and without triton permeabilization. A clear and specific immune reaction was detected only in cells expressing AQP1 (merge signal in orange, top right panel). Absence of IgG anti-AQP1 was revealed in all rest of conditions tested: with an anti-human IgG secondary antibody; with human serum from a patient with NMOSD (+NMOSD); and with human serum from a patient negative for NMOSD (−NMOSD). Merge of fluorescence signal can only be seen when the commercial antibody for AQP1 was used (orange/yellow signal, top right panel). Scale bar = 15 µm.

As seen in Figure 1 and Figure 2, an apparently normal expression of the fluorescent protein hAQP1-EGFP was being produced in the transfected HEK cells. However, to further discard the possibility that AQP1 was not being inserted in an appropriate way into the plasma membrane of cells, hindering the natural recognition by antibodies which could explain the lack of immune reaction and consequent detection of AQP1 antibodies, we performed experiments using a commercial primary antibody for AQP1. Results from these analyses are shown in Figure 3.

A red fluorescent signal with the IgG-antihuman AQP1 commercial was observed clearly labeling all cells expressing hAQP1-EGFP and stained in green, resulting in a perfect overlapping merge of fluorescence signals. Thereby, these results demonstrate two important findings: First, that expression of AQP1 in an antigenic way occurs in HEK cells and second, that human serums either from the NMOSD subjects or not, did not present IgG anti-AQP1 or at least not enough levels, to allow differentiation among these patients by our CBA procedure. We conclude then that the absence of anti-AQP1 antibodies observed in all the 205 serums analyzed with the CBA assay is not due to the lack of AQP1 expression in the cells, but instead is likely due to a true absence of these antibodies in the serum, at least at levels detectable with our protocol of CBA.

A previous study [20], using a live CBA assay with a protocol that allowed authors for a nice detection of AQP4 antibodies in human serum, also failed to confirm the presence of AQP1 antibodies in NMOSD in agreement with results presented here by us. As indicated by Schanda et al., [20] we also agree that AQP1 antibodies in patients must react against intracellular epitopes of AQP1, otherwise patients would suffer of severe anemia due to the hemolytic action of antibodies reacting over the Colton blood group antigen present on the erythrocytes membrane, and that is not the case in any of our patients. Alternative conformational ways for expression of AQP1 in astrocytes compare to the way the protein is expressed in HEK293 cells, as indicated by Schanda et al., [20] could still offer some explanation for the lack of positive results when using CBA assays for detection of AQP1 antibodies, but all these explanations will need further experimental analysis using different experimental approaches.

**Figure 4:**
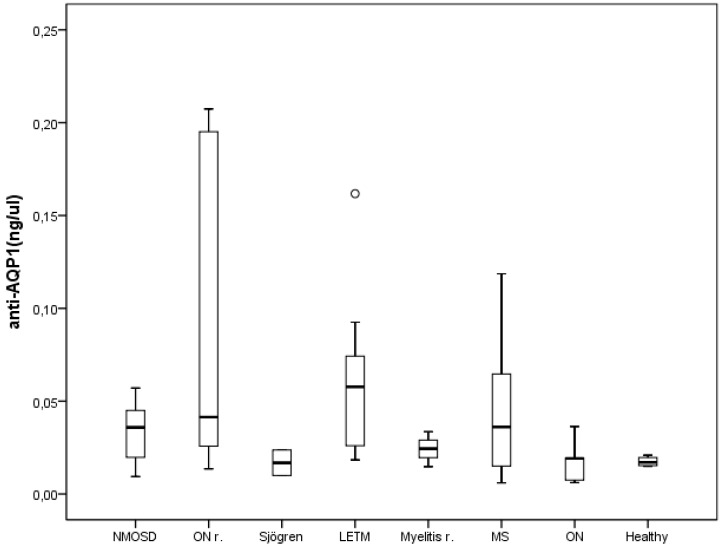
Detection by ELISA assay of anti-AQP1 antibodies in serum of patients. Serums from different pathologies according to the classification shown in Table 1 were analyzed including a group with the NMO spectrum disorder (+NMOSD). Abbreviations of pathologies are as indicated in Table 1. Insignificant differences were obtained among groups by analysis of variance for nonparametric data using the Kruskal–Wallis test (*p* = 0.067). Medians and interquartile range are represented and the number of patients analyzed per group were as follows: 8 NMOSD; 6 ON r. (Optic neuritis recurrent); 2 Sjörgen; 9 LETM (Longitudinally extensive transverse myelitis); 3 Myelitis repetition; 18 MS (Multiple sclerosis); 5 ON (Optic neuritis); 4 Control (Healthy). Circle, correspond to an outsider data point.

In that sense, we carried out experiments of ELISA to further clear this apparent discrepancy in the field and, although in few serums values of anti-AQP1 antibodies were detected (Figure 4), the overall comparative analysis of the levels of anti-AQP1 antibodies among all groups analyzed revealed no statistical differences among them, making impossible at this state for us any discussion about presence of AQP1 antibodies in those serums. Therefore, our findings with the ELISA are inconsistent with an association between presence of anti-AQP1 antibodies and NMOSD pathology. We are aware that a larger number of samples in our analysis would be desirable to increase the power of our conclusions, and for allowing us determination of the cut-off level of anti-AQP1 antibody in ELISA, but such aim was unfortunately impossible at the time of the study. Our general impression, given these results, is that AQP1 antibodies does not represent another specific biomarker of the NMOSD but its rare presence in serum of some patients as indicated by other authors [17,18,19] may probably be associated with an increased autoimmune humoral response as previously observed in patients with MS in which presence of antinuclear (ANA), anticardiolipin (ACA) and anti-Ro (SS-A) antibodies have been detected [22,23]. Like these antibodies, the presence of anti-AQP1 antibodies may be indicative of an underlying autoimmune disease; but more than associated to particular aspects of any of these diseases, it appears as an epiphenomenon of a more diffuse immunological dysfunction. Nevertheless, further analysis with the ELISA, or other immune assays, especially those with detection of the protein in suspension, accompanied with more analysis including larger number of patients on each subgroup of these diverse pathologies as well as more cases of the NMOSD would be necessary to strength our incipient hypothesis.

## 3. Materials and Methods

### 3.1. Subjects and Serum Recollection

The study includes 205 subjects (135 female) classified into 7 groups based on their medical diagnosis (Table 1). Group 1 comprised 8 patients with NMOSD according to the current diagnostic criteria [12]. Group 2 consisted of 94 patients with MS according to 2010-reviewed McDonald criteria [24]. In relation to the MS course, we further divided this group into 85 patients with remitting-relapsing MS, 7 with primary progressive MS and 2 with secondary progressive form. Group 3 was composed of 39 patients with optica neuritis. Twenty-nine patients with myelitis formed group 4. Concerning to the length of the spinal cord lesion, we distinguished between patients whose lesions extended more than 3 vertebral segments (longitudinally extensive myelitis) and those with spinal plaques extended up to 3 vertebral segments (10 and 16 patients, respectively). Besides, we categorized the patients of the latter two groups depending whether they suffered an isolated episode or several relapses. Group 5 comprised 9 patients with other demyelinating idiopathic disorders of the CNS and group 6 formed by subjects with other neurological disorders, both groups described in Table 1. Besides the MRI (magnetic resonance imaging) scans and the cerebrospinal fluid analysis, peripheral blood exam that included blood count, biochemistry, erythrocyte sedimentation rate, vitamins, thyroid hormones, long chain fatty acids, angiotensin converting enzyme, immunological and serological examinations were conducted in all patients in order to exclude alternative etiologies to their final diagnosis. Finally, group 7 was composed of 8 healthy controls.

### 3.2. Plasmid Construction, Cell Culture and Cell Transfection

The pCMV6-AC-AQP1-GFP (human AQP1-GFP) was purchased from OriGene Technologies Inc. (Rockville, MD, USA) as a plasmid ready to use in mammalian cells and the human M23-AQP4 isoform was amplified by PCR from the commercial vector pDNR-LIB cDNA (Takara Bio Europe/Clontech, Saint-Germain-en-Laye, France) using specific primers and cloned into pEGFP-N1 (Takara), for transfection and expression in the HEK293T cell line as previously described [20]. Both AQP-EGFP constructs allow synthesis of either AQP1 or AQP4 fusion fluorescent proteins with the enhanced-green-fluorescent-protein-EGFP bound to its carboxyl ends. HEK293T cells were cultured in DMEM with 10% FCS and 1% penicillin/streptomycin (37 °C, 5% CO_2_). Cells (2 × 10^5^) were seeded in 35 mm dishes for transfection with Lipofectamine 2000 (Invitrogen, Carlsbad, CA, USA) as described before [21,25].Transfected cells were maintained for 25–30 passages until fluorescence signal of AQPs expression decrease below 90%.

### 3.3. Immunofluorescence Assay

Based in a method described previously we evaluated presence of AQP1- and AQP4-Abs in the serum of patients using a protocol that we called basic assay [21]. The protocol combines expression of a fusion green fluorescent protein (AQP1- or AQP4-EGFP) with the use of a red fluorescent goat anti-human secondary antibody that, by dual labeling (green and red fluorescence), constitutes a method with extremely high sensitivity and specificity to identify positive patients for any of those antibodies. Briefly, 24 h before starting the immune-assay, HEK293T plated cells at about 80% of confluence were transfected with either AQP1- or AQP4-EGFP constructs. Then two different pretreatments were performed separately, one that we called “permeabilization” protocol, and another one called “without permeabilization”, equivalent to the original protocol described initially [21]. In the permeabilization protocol the cells were fixed with paraformaldehyde 4% (5 min) and then washed with triton X-100 2% (SIGMA, St. Louis, MO, USA) in PBS (PBTx, 10 min), before incubating (1 h) in FCS 10% with 1 mg/mL BSA in PBTx for the blocking step. Afterward, incubation with the serum’s patient (1:10 dilution for detection of anti-AQP1 and 1:50 dilution for detection of anti-AQP4, 1 h at room temperature) or with an anti-Aquaporin 1 antibody (ab 117970, ABCAM, Cambridge, UK) (1:500 dilution) raised against a full length recombinant human Aquaporin 1 produced in HEK293T cells, was followed by three times washes with PBS and then 30 min of incubation with Alexa Fluor 568 goat anti-human secondary antibody (Invitrogen, Carlsbad, CA, USA). In the method in which the cells were not permeabilized, the Triton X-100 was removed from each step. So, after fixing the cells with paraformaldehyde 4% (5 min), a washing step in PBS of 5 min was followed for a blocking period (1 h) in 10% FCS with 1 mg/mL BSA in PBS, and finally the cells were fixed (1 min), after the secondary antibody incubation, with a mixture of ethanol 95% and acetic acid 5%. Nuclei were stained with 4′,6′-diamidino-2-phenylindole (DAPI, 1:1000) and a Leica DM IRBE confocal microscope (Leica, Wetzlar, Germany) was used to observe the slides. Five photos (40×) per sample were randomly taken and the NIH ImageJ software (NIH, Bethesda, MD, USA) used for densitometry analysis of fluorescence.

### 3.4. ELISA for AQP1 (Aquaporin-1)

#### 3.4.1. Preparation of AQP1 Protein Homogenate

HEK293T cells were transfected with a pcDNA3-AQP1 for expression of human AQP1 protein as previously used [26,27]. After 24–48 h of transfection cells were washed with cold PBS and treated with trypsin 0.25% (GIBCO, Paisley, UK) for collection. The cell pellet was resuspended in 1 mL of cold PBS and centrifuged at 300× *g* for 5 min at 4 °C. For whole-cell protein extract, pellet was dissolved in 500 μL of lysis buffer: 137 mM NaCl, 20 mM Tris (pH: 8); 1% IGEPAL-CA630 (Sigma Aldrich, St. Louis, MO, USA), a nonionic, non-denaturing detergent; 10% Glycerol and 10 μL/mL of complete protease inhibitors cocktail (Sigma Aldrich). The homogenate was left on ice 15 min, vortex, and then centrifuged at 16,000× *g* for 15 min at 4 °C, and extracted proteins remain in the supernatant. Protein concentration was analyzed with the Bradford method (BioRad Protein Assay, BioRad, Berkeley, CA, USA) and kept at −20 °C until loading into plates for ELISA assay.

#### 3.4.2. Adhesion of AQP1 Protein for ELISA Assay

General guidelines for ELISA assay have been described elsewhere [28]. Proteins prepared as before were diluted at 20 μg/mL final concentration in 0.01 M buffer carbonate and 50 μL per well of protein suspension were loaded into a 96 well plate for ELISA (Microwell MaxiSorp, Nunc, Waltham, MA, USA), afterwards the plate was covered with a plastic film and left overnight at 4 °C. The next day the solution was removed and the plate washed three times by filling the wells with 200 μL PBS1X + 0.05% Tween and once with PBS1X.

Blocking: To block the remaining protein-binding sites in the coated wells 200 μL of SuperBlock Blocking Buffer (ThermoScientific, Vantaa, Finland) were added per well and incubated at room temperature for 1 h, maintaining the plate cover with plastic film. Then, blocking solution was removed and the plate was washed three times by filling the wells again with 200 μL PBS1X + 0.05% Tween and once with PBS1X.

#### 3.4.3. Incubation with Primary and Secondary Antibodies

Two primary antibodies, 100 μL per well, were used; a commercial antibody anti-AQP1 (ab15080, ABCAM) diluted 1:10,000 in PBS with 2% BSA, that serves as a control to set the assay conditions, and the patient serums without dilution. The incubation was allowed to proceed over night at 4 °C and the next day plates were washed as indicated for removing the blocking solution mentioned above. Then, incubation with the secondary antibodies for 1 h at room temperature was carried out. Horseradish peroxidase conjugated goat anti-rabbit IgG antibody diluted (1:5000) in PBS with 2% BSA for the AQP1 commercial antibody, and horseradish peroxidase conjugated chicken anti-human IgG antibody for the patient serum antibodies were used. Wash of plates at the end was again carried out as before.

#### 3.4.4. Signal Detection: Per Well, 100 μL of 3,3′,5,5′-Tetramethylbenzidine (TMB)

TMBOne solution (Promega, Madison, WI, USA) was added and incubated at room temperature for 15 min to allow enzymatic reaction and developing of colored substrate. Then, 100 μL of HCl 1N were added per well to stop the reaction and absorbance at 450 nm was measured in a plate reader system (Multiskan Spectrum-Thermo, Vantaa, Finland).

### 3.5. Statistical Analysis

Data are presented as mean ± standard error of the mean, and analyzed using the Statistical Package for Social Sciences (SPSS Inc., Chicago, IL, USA), version 19.0. Data with a non-normal distribution were analyzed using analysis of variance (ANOVA) for non-parametric data with the Kruskal–Wallis H test.

## 4. Conclusions

Our study does not show sustained detection of anti-AQP1 in serum of NMOSD patients analyzed by our fixed cell based assay or ELISA protocol. To our understanding, these antibodies do not seem to allow confirmation of specific immune disorders associated with NMOSD.

## Figures and Tables

**Table 1:** Demographic and clinical variables of 205 patients.

Diagnosis	Number of Patients (205)	Gender Female/Male	Mean Age at Inclusion ± SD (range)	AQP4+ Antibodies	AQP1+ Antibodies
1. NMOSD	8	7/1	57.14 ± 13.52 (40–80)	6	0
2. MS	94	66/28	39.87 ± 11.84 (18–76)	0	0
* RRMS	85	59/26		0	0
* PPMS	7	5/2		0	0
* SPMS	2	2/0		0	0
3. Idiopathic ON	39	27/12	39.55 ± 13.02 (14–68)	0	0
* Isolated episode	30	22/8		0	0
* Recurrent idiopathic ON	9	5/4		0	0
4. Idiopathic myelitis	29	19/10	45.13 ± 13.58 (21–69)	0	0
* Isolated episode:	26	17/9		0	0
>3 vertebral segments	10	5/5		0	0
<3 vertebral segments	16	12/4		0	0
* Recurrent idiopathic myelitis	3	2/1		0	0
5. OIDD of the CNS	9	5/4	48.88 ± 10.37 (26–60)	0	0
* ADEM	2	0/2		0	0
* Infratentorial CIS	4	2/2		0	0
* RIS	3	3/0		0	0
6. Other neurology disorders	18	8/10	51.35 ± 12.79 (26–79)	0	0
* Myelitis associated with lupus	3	3/0		0	0
* Myelitis associated with sarcoidosis	1	0/1		0	0
* ON associated with Sjögren syndrome	1	1/0		0	0
* Multifocal motor neuropathy	3	0/3		0	0
* CIDP	1	1/0		0	0
* Hereditary spastic paraparesis	1	1/0		0	0
* Spinal infraction	2	1/1		0	0
* Ischemic optic neuropathy	6	1/5		0	0
7. Healthy controls	8	6/2	36.42 ± 8.12 (27–47)	0	0

AQP: Aquaporin; CNS: central nervous system; SD: Standard deviation; NMOSD: Neuromyelitis optica syndrome disorder; ON: Optic neuritis; MS: Multiple sclerosis; RRMS: Remitting relapsing multiple sclerosis; SPMS: Secondary progressive multiple sclerosis; PPMS: Primary progressive multiple sclerosis; ADEM: Acute disseminated encephalomyelitis; CIDP: Chronic inflammatory demyelinating polyneuropathy, CIS: Clinically isolated syndrome, RIS: Radiological isolated syndrome; OIDD: Other idiopathic demyelinating disorders of the CNS. * correspond to further division of the MS group.

## References

[1] Wingerchuk D.M., Lennon V.A., Lucchinetti C.F., Pittock S.J., Weinshenker B.G. (2007). The spectrum of neuromyelitis optica. Lancet Neurol..

[2] Uzawa A., Mori M., Kuwabara S. (2014). Neuromyelitis optica: Concept, immunology and treatment. J. Clin. Neurosci..

[3] Lennon V.A., Wingerchuk D.M., Kryzer T.J., Pittock S.J., Lucchinetti C.F., Fujihara K., Nakashima I., Weinshenker B.G. (2004). A serum autoantibody marker of neuromyelitis optica: Distinction from multiple sclerosis. Lancet.

[4] Pittock S.J., Weinshenker B.G., Lucchinetti C.F., Wingerchuk D.M., Corboy J.R., Lennon V.A. (2006). Neuromyelitis optica brain lesions localized at sites of high aquaporin 4 expression. Arch. Neurol..

[5] Weinshenker B.G., Wingerchuk D.M. (2008). Neuromyelitis optica: Clinical syndrome and the NMO-IgG autoantibody marker. Curr. Top. Microbiol. Immunol..

[6] Amiry-Moghaddam M., Hoddevik E.H., Ottersen O.P. (2010). Aquaporins: Multifarious roles in brain. Neuroscience.

[7] Venero J.L., Vizuete M.L., Ilundain A.A., Machado A., Echevarria M., Cano J. (1999). Detailed localization of aquaporin-4 messenger rna in the CNS: Preferential expression in periventricular organs. Neuroscience.

[8] Verkman A.S., Phuan P.W., Asavapanumas N., Tradtrantip L. (2013). Biology of AQP4 and anti-AQP4 antibody: Therapeutic implications for NMO. Brain Pathol..

[9] Cayrol R., Saikali P., Vincent T. (2009). Effector functions of antiaquaporin-4 autoantibodies in neuromyelitis optica. Ann. N. Y. Acad. Sci..

[10] Fujihara K. (2011). Neuromyelitis optica and astrocytic damage in its pathogenesis. J. Neurol. Sci..

[11] Lucchinetti C.F., Mandler R.N., McGavern D., Bruck W., Gleich G., Ransohoff R.M., Trebst C., Weinshenker B., Wingerchuk D., Parisi J.E. (2002). A role for humoral mechanisms in the pathogenesis of Devic’s neuromyelitis optica. Brain.

[12] Wingerchuk D.M., Banwell B., Bennett J.L., Cabre P., Carroll W., Chitnis T., de Seze J., Fujihara K., Greenberg B., Jacob A. (2015). International consensus diagnostic criteria for neuromyelitis optica spectrum disorders. Neurology.

[13] Melamed E., Levy M., Waters P.J., Sato D.K., Bennett J.L., John G.R., Hooper D.C., Saiz A., Bar-Or A., Kim H.J. (2015). Update on biomarkers in neuromyelitis optica. Neurol. Neuroimmunol. Neuroinflamm..

[14] Badaut J., Brunet J.F., Grollimund L., Hamou M.F., Magistretti P.J., Villemure J.G., Regli L. (2003). Aquaporin 1 and aquaporin 4 expression in human brain after subarachnoid hemorrhage and in peritumoral tissue. Acta Neurochir. Suppl..

[15] Oshio K., Binder D.K., Liang Y., Bollen A., Feuerstein B., Berger M.S., Manley G.T. (2005). Expression of the aquaporin-1 water channel in human glial tumors. Neurosurgery.

[16] Suzuki R., Okuda M., Asai J., Nagashima G., Itokawa H., Matsunaga A., Fujimoto T., Suzuki T. (2006). Astrocytes co-express aquaporin-1, -4, and vascular endothelial growth factor in brain edema tissue associated with brain contusion. Acta Neurochir. Suppl..

[17] Tzartos J.S., Stergiou C., Kilidireas K., Zisimopoulou P., Thomaidis T., Tzartos S.J. (2013). Anti-aquaporin-1 autoantibodies in patients with neuromyelitis optica spectrum disorders. PLoS ONE.

[18] Tuzun E., Tzartos J., Ekizoglu E., Stergiou C., Zisimopoulou P., Coban A., Shugaiv E., Turkoglu R., Kurtuncu M., Baykan B. (2014). Aquaporin-1 antibody in neuromyelitis optical patients. Eur. Neurol..

[19] Long Y., Zheng Y., Shan F., Chen M., Fan Y., Zhang B., Gao C., Gao Q., Yang N. (2014). Development of a cell-based assay for the detection of anti-aquaporin 1 antibodies in neuromyelitis optica spectrum disorders. J. Neuroimmunol..

[20] Schanda K., Waters P., Holzer H., Aboulenein-Djamshidian F., Leite M.I., Palace J., Vukusic S., Marignier R., Berger T., Reindl M. (2015). Antibodies to aquaporin-1 are not present in neuromyelitis optica. Neurol. Neuroimmunol. Neuroinflamm..

[21] Sanchez Gomar I., Diaz Sanchez M., Ucles Sanchez A.J., Casado Chocan J.L., Ramirez-Lorca R., Serna A., Villadiego J., Toledo-Aral J.J., Echevarria M. (2014). An immunoassay that distinguishes real neuromyelitis optica signals from a labeling detected in patients receiving natalizumab. BMC Neurol..

[22] De Andres C., Guillem A., Rodriguez-Mahou M., Lopez Longo F.J. (2001). Frequency and significance of anti-Ro (SS-A) antibodies in multiple sclerosis patients. Acta Neurol. Scand..

[23] Szmyrka-Kaczmarek M., Pokryszko-Dragan A., Pawlik B., Gruszka E., Korman L., Podemski R., Wiland P., Szechinski J. (2012). Antinuclear and antiphospholipid antibodies in patients with multiple sclerosis. Lupus.

[24] Polman C.H., Reingold S.C., Banwell B., Clanet M., Cohen J.A., Filippi M., Fujihara K., Havrdova E., Hutchinson M., Kappos L. (2011). Diagnostic criteria for multiple sclerosis: 2010 revisions to the Mcdonald criteria. Ann. Neurol..

[25] Abreu-Rodriguez I., Sanchez Silva R., Martins A.P., Soveral G., Toledo-Aral J.J., Lopez-Barneo J., Echevarria M. (2011). Functional and transcriptional induction of aquaporin-1 gene by hypoxia; analysis of promoter and role of Hif-1α. PLoS ONE.

[26] Galan-Cobo A., Ramirez-Lorca R., Toledo-Aral J.J., Echevarria M. (2016). Aquaporin-1 plays important role in proliferation by affecting cell cycle progression. J. Cell. Physiol..

[27] Serna A., Galan-Cobo A., Rodrigues C., Sanchez-Gomar I., Toledo-Aral J.J., Moura T.F., Casini A., Soveral G., Echevarria M. (2014). Functional inhibition of aquaporin-3 with a gold-based compound induces blockage of cell proliferation. J. Cell. Physiol..

[28] Villadiego J., Mendez-Ferrer S., Valdes-Sanchez T., Silos-Santiago I., Farinas I., Lopez-Barneo J., Toledo-Aral J.J. (2005). Selective glial cell line-derived neurotrophic factor production in adult dopaminergic carotid body cells in situ and after intrastriatal transplantation. J. Neurosci..

